# 24 h-accelerometry in epidemiological studies: automated detection of non-wear time in comparison to diary information

**DOI:** 10.1038/s41598-017-01092-w

**Published:** 2017-05-22

**Authors:** Lina Jaeschke, Agnes Luzak, Astrid Steinbrecher, Stephanie Jeran, Maike Ferland, Birgit Linkohr, Holger Schulz, Tobias Pischon

**Affiliations:** 1 Molecular Epidemiology Group, Max Delbrueck Center for Molecular Medicine in the Helmholtz Association (MDC), Berlin, Germany; 2 Institute of Epidemiology I, Helmholtz Zentrum München - German Research Center for Environmental Health, Neuherberg, Germany; 3 Institute of Epidemiology II, Helmholtz Zentrum München - German Research Center for Environmental Health, Neuherberg, Germany; 4 Comprehensive Pneumology Center Munich (CPC-M), Member of the German Center for Lung Research, Munich, Germany; 5 Charité – Universitätsmedizin Berlin, Berlin, Germany; 6 DZHK (German Center for Cardiovascular Research), partner site Berlin, Germany

## Abstract

Estimation of physical activity using 24 h-accelerometry requires detection of accelerometer non-wear time (NWT). It is common practice to define NWT as periods >60 minutes of consecutive zero-accelerations, but this algorithm was originally developed for waking hours only and its applicability to 24 h-accelerometry is unclear. We investigated sensitivity and specificity of different algorithms to detect NWT in 24 h-accelerometry compared to diary in 47 ActivE and 559 KORA participants. NWT was determined with algorithms >60, >90, >120, >150, or >180 minutes of consecutive zero-counts. Overall, 9.1% (ActivE) and 15.4% (KORA) of reported NWT was >60 minutes. Sensitivity and specificity were lowest for the 60-min algorithm in ActivE (0.72 and 0.00) and KORA (0.64 and 0.08), and highest for the 180-min algorithm in ActivE (0.88 and 0.92) and for the 120-min algorithm in KORA (0.76 and 0.74). Nevertheless, when applying these last two algorithms, the overlap of accelerometry with any diary based NWT minutes was around 20% only. In conclusion, only a small proportion of NWT is >60 minutes. The 60-min algorithm is less suitable for NWT detection in 24 h-accelerometry because of low sensitivity, specificity, and small overlap with reported NWT minutes. Longer algorithms perform better but detect lower proportions of reported NWT.

## Introduction

Physical activity reduces the risk of chronic diseases and premature death^1–3^. Assessing physical activity in humans in epidemiological studies is challenging, and has mostly relied on self-reported questionnaires with low to moderate validity as compared to objective measures^4, 5^. Today, by measuring the body’s acceleration in all three spatial axes, accelerometers enable an improved physical activity assessment under free-living conditions. While, most previous studies use accelerometry assessed during waking hours only^6–9^, 24 h-accelerometry over multiple days is now being used in several large cohort studies like the German National Cohort, allowing to capture a comprehensive view on the individual habitual physical activity^10, 11^.

One important premise for the interpretation of accelerometer data in terms of habitual physical activity is knowledge about whether or not the device has been worn appropriately. In this context, detection of accelerometer non-wear time (NWT) is essential to correctly classify habitual physical activity. Assessment of NWT using diaries or questionnaires is time consuming for study participants and researchers, and may therefore not be feasible in large-scale observational studies. Automated detection from accelerometry data may be an efficient alternative way for assessment of NWT. Most previous studies have defined NWT as periods of >60 consecutive minutes of zero-acceleration readings^6, 12–14^. However, this definition, based on an algorithm proposed by Troiano *et al*., was developed using uniaxial accelerometers worn during waking hours only^9, 15^. While some previous studies already indicated that definitions with longer periods of zero-acceleration readings might more accurately detect NWT during waking hours^16–18^, complexity is added when recordings are conducted over 24 h periods and thus include periods of sleeping, since in sleeping phases motionless periods might be longer than 60 minutes. It is thus unclear to what extent the 60-min algorithm is suitable to detect NWT in accelerometry data captured over 24 h periods.

The aim of this study was therefore to assess to what extent algorithms of periods of >60 minutes of acceleration zero-readings are appropriate to define NWT in 24 h-accelerometry data assessed over multiple days in the general adult population. This was accomplished in two epidemiological studies on adults from the North (ActivE, Berlin; convenience sample) and the South of Germany (KORA, Augsburg; population-based sample).

## Methods

### Study populations

We used cross-sectional data of the ActivE study (Berlin, Germany) and the KORA (KORA - Cooperative Health Research in the Region of Augsburg, Germany) FF4 cohort. The ActivE study originally aimed to quantify activity-related energy expenditure using 24 h-accelerometry over two weeks. A convenience sample of 50 participants was recruited aged 20–69 years with body-mass index (BMI) 18.5–35.0 kg/m². Participants with physiological conditions interfering with energy metabolism or weight stability as well as mobility impairments were excluded. The initial KORA S4 cohort included 4,261 participants aged 25–74 years from the general population in the area of Augsburg, Germany, recruited between 1999–2001^16^. For the present analysis, data was used from the second follow-up, KORA FF4 (in the following referred to as ‘KORA’), which was performed in 2013–2014, enrolling 2,279 participants. Of those, 1,043 participants were asked to take part in the 24 h-accelerometry; 562 agreed to participate. The study protocols of ActivE and KORA were approved by the ethics committee of the Charité - Universitätsmedizin Berlin and the Bavarian Medical Association, respectively, and by the local data protection officers. All investigations were carried out in accordance with the relevant guidelines and regulations. All participants gave written informed consent.

### Data collection

At the study centers, anthropometric measurements were taken, and information on socio-demographic, economic, and lifestyle factors was collected. Participants were provided with the triaxial accelerometer ActiGraph GT3X+ (ActivE) or GT3X (KORA) (both ActiGraph LLC, Pensacola, FL, USA), and were asked to wear the device for a period of two weeks (ActivE) and one week (KORA) for all waking and sleeping phases except for water activities, sauna, or high contact sports. Previous studies have shown good validity and reliability of the ActiGraph GT3X+ and GT3X for assessment of habitual physical activity^17–19^. The only difference between the GT3X+ and the GT3X is that the former is waterproof, whereas the latter is not. In ActivE, accelerometers were put on during the first study visit, and participants were instructed to wear the device on the right hip. Because of limited recording capability, each participant was provided with a second pre-initialized accelerometer to be worn during the second week and data of both devices were later on joined to have two weeks of 24 h-accelerometry. In KORA, participants started wearing the accelerometer the morning after the study visit, and they were instructed to wear the accelerometer at the hip on the side of the dominant hand during daytime and to move it from hip to the wrist of the non-dominant hand overnight^20^. All participants kept activity diaries to record sleep/wake phases and starting/ending time and reasons of any NWT period. Due to missing or inconsistent diary or accelerometry data three ActivE and three KORA participants were excluded from all analyses, resulting in 47 ActivE and 559 KORA participants included in the present analysis.

The ActiLife software was used to initialize accelerometers and to download activity data as ‘counts’ (ActivE study, version 6.11.0; KORA study, version 6.11.2; ActiGraph LLC, Fort Walton Beach, FL, USA). Raw accelerometry data were sampled by a 12 bit analog to digital converter (dynamic range; ActivE: ±6G, KORA: ±3G) at a constant 30 (KORA, stored at a 1 Hz rate) or 100 Hz (ActivE, stored at a 100 Hz rate) rate using all three spatial axes (filter set to default, ‘normal’, in both studies). Data were converted into 60 sec-epochs and extracted as ‘vector magnitude counts/minute’ (resulting from acceleration detected via the vertical, horizontal, and perpendicular axis).

### Statistical analysis

Age, height, weight, BMI, NWT minutes, and number of NWT periods are presented as median and interquartile range (IQR). NWT parameters were averaged per participant over the total time of accelerometry recorded.

**Figure 1:**
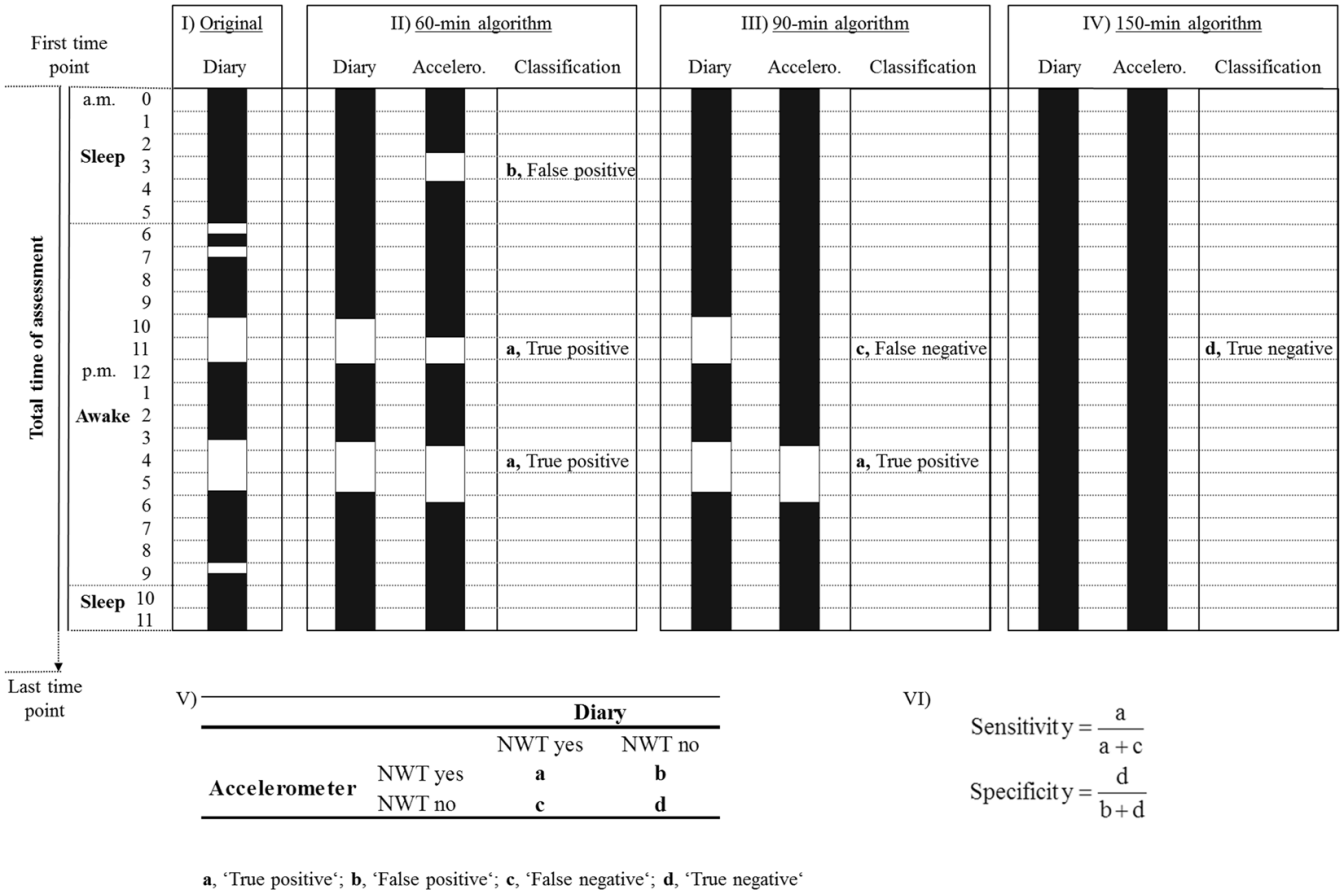
Exemplary description of non-wear time (NWT) validation using consecutive time periods for calculation of sensitivity and specificity of NWT algorithms to detect NWT periods >60 to >180 minutes (black rectangles, wear time based on 24 h-accelerometry or diary data, respectively; white rectangles, NWT based on 24 h-accelerometry or diary data, respectively). As an example, in the original diary data (panel I), five NWT periods during waking were reported being 30, 30, 115, 130, and 30 minutes, respectively. When applying the 60-min NWT algorithm (panel II), two of all NWT periods in diary (115 and 130 minutes, respectively); at the same time, three NWT periods occurred based on the 24 h-accelerometry data (accelero.) during waking (65 and 140 minutes, respectively) and sleeping (75 minutes). Using the 90-min NWT algorithm (panel III), two NWT periods in diary were still detected (115 and 130 minutes, respectively); based on accelerometry, one NWT period was detected (140 minutes). No NWT was detected in diary or accelerometry data when applying the 150-min algorithm (panel IV). For each NWT algorithm, detected NWT periods were classified according to the fourfold table in panel V. Sensitivity (proportion of true positively identified NWT) and specificity (proportion of true negatively identified NWT) were then calculated using the formulas in panel VI.

For each participant we determined (a) the total time of assessment as period between first and last recorded time point of the diary, and (b) each waking and sleeping phase of the measured days/nights over the study period as recorded in the diary. Thus, we were able to compare NWT between accelerometry and diary over the total time of assessment and separately over waking or sleeping phases (Fig. 1). Three approaches were used to compare NWT detected based on diary and accelerometry data:

1. *Sensitivity and specificity of NWT algorithms* >*60 to* >*180* *minutes based on accelerometry compared to diary*.

We determined each participant’s NWT based on the diaries’ information by identifying consecutive time periods of reported NWT between >60 to >180 minutes, respectively. To derive NWT based on accelerometry data, we used the extracted data of the ‘vector magnitude counts/minute’ and determined periods of >60, >90, >120, >150, or >180 minutes of consecutive acceleration zero-counts without allowing for interruptions. Based on the different algorithms, each identified NWT period was compared between accelerometry and diary, with the diary set as reference. If there was an overlap in length of NWT periods between accelerometry and diary of ≥50% this was coded as true positively classified NWT (Fig. 1, panel I–VI, classification ‘a, true positive’). If the overlap was <50%, the period was recorded as ‘not assigned’ (not included in analyses of sensitivity and specificity). If a NWT period in the accelerometry data did not correspond to a diary period, this period was assigned as false positively classified (Fig. 1, panel I–VI, classification ‘b, false positive’). A NWT period detected in the diary that was not detected in the accelerometry data was assigned as false negative (Fig. 1, panel I–VI, classification ‘c, false negative’). In case both, the diary and the accelerometry data indicated a continuous wear time during the period considered for analysis, it was classified as true negative (Fig. 1, panel I–VI, classification ‘d, true negative’). Based on the total number of NWT periods identified, sensitivity (proportion of true positively identified NWT) and specificity (proportion of true negatively identified NWT) were calculated for each NWT algorithm (Fig. 1, panel I–VI)^21^.

2. *Overlap of accelerometry and diary NWT periods identified using the* >*60-min to* >*180-min algorithms*.

**Figure 2:**
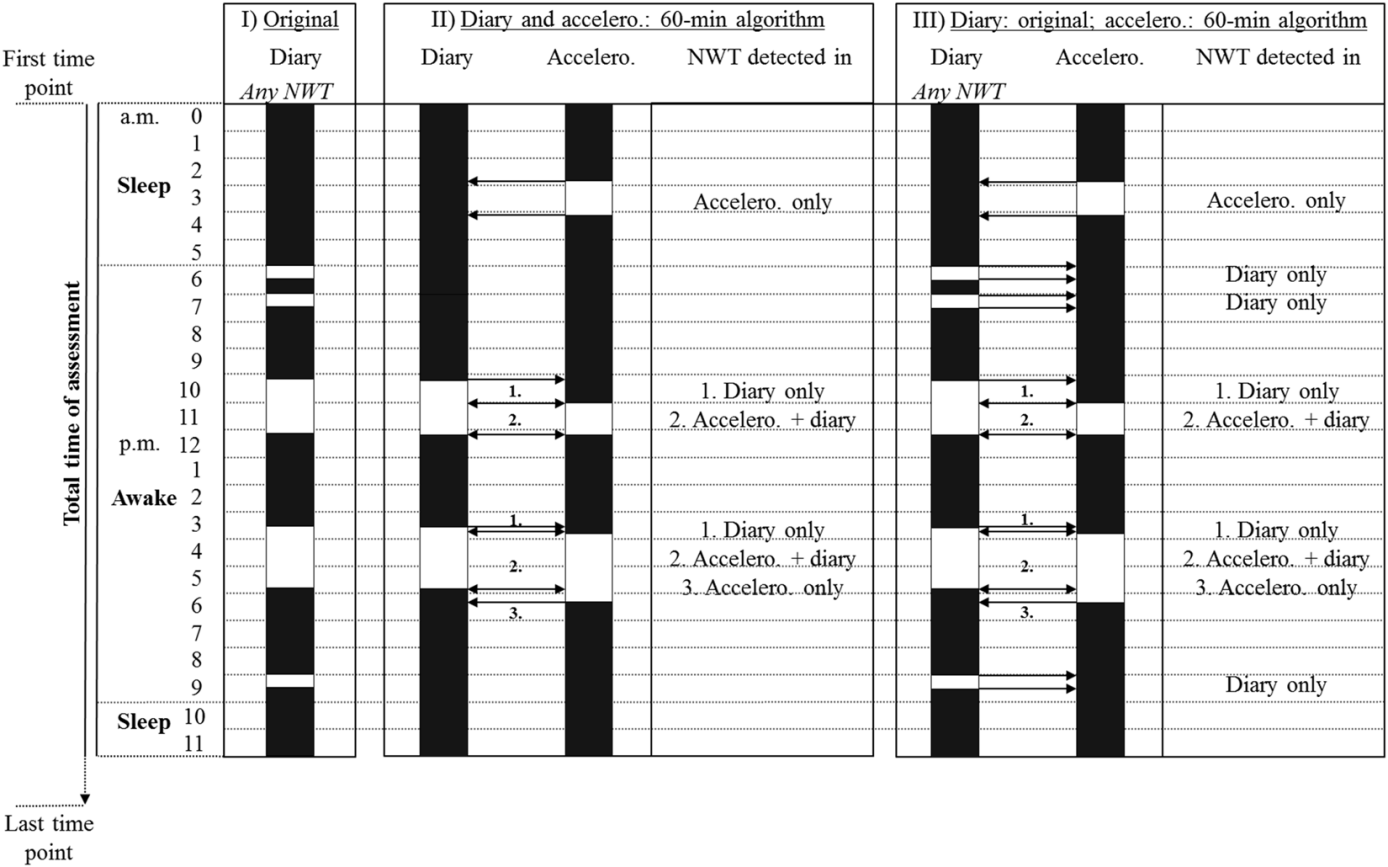
Exemplary description of non-wear time (NWT) validation using a minute-by-minute evaluation for calculation of overlap in length (minutes) of NWT detected based on accelerometry (accelero.) as compared to diary (black rectangles, wear time based on 24 h-accelerometry or diary data, respectively; white rectangles, NWT based on 24 h-accelerometry or diary data, respectively). As an example, in the original diary data (panel I), five NWT periods during waking were reported being 30, 30, 115, 130, and 30 minutes, respectively. When applying the 60-min NWT algorithm (panel II) to both accelerometry and diary data two of all NWT periods in diary (115 and 130 minutes, respectively), and three NWT periods in accelerometry were detected (75, 65, and 140 minutes, respectively). When applying the 60-min NWT algorithm (panel III) to accelerometry data only while *any* NWT regardless of a minimal length reported in the diary was assessed, still three NWT periods in accelerometry (75, 65, and 140 minutes, respectively) but all five ‘original’ NWT periods in diary (30, 30, 115, 130, and 30 minutes, respectively) were detected. For each NWT algorithm and both approaches (panel II and III), NWT minutes detected by *accelerometry only*, in *diary only* and in both *accelerometry and diary* were assessed. We then calculated the relative contribution of each of these to the potential total NWT (i.e., NWT detected in either *diary*, *accelerometry*, or *both*). Overlap was defined as the NWT detected in both *accelerometry and diary*.

We determined the overlap of the detected NWT between accelerometry and diary on a minute-by-minute basis for each NWT algorithm for NWT-periods >60 minutes (Fig. 2). We calculated for each algorithm the length (minutes) of: (1) NWT >60 minutes detected in both, *diary and accelerometry* data (Fig. 2, panel II-III, case ‘accelero. +diary’), (2) NWT >60 minutes detected in *diary only* (Fig. 2, panel II-III, case ‘diary only’), and, (3) NWT >60 minutes detected by *accelerometry only* (Fig. 2, panel II-III, case ‘accelero. only’). We then calculated the relative contribution of the sum of NWT minutes in NWT periods >60 minutes identified as case 1 (overlap, i.e., NWT minutes detected in both, *diary and accelerometry*), case 2 (*diary only*), and case 3 (*accelerometry only*), to the potential total NWT which is NWT detected by either *diary*, *accelerometry*, or *both*.

3. *Overlap of any diary NWT with accelerometry NWT identified using the* >*60-min to* >*180-min algorithms*.

To investigate the ability of the algorithms to detect NWT at all and to assess the contribution of NWT periods <60 minutes to total NWT in 24 h-accelerometry data, analogue analyses to the second approach were performed comparing *any* NWT detected in the diary (regardless of a minimal length) with NWT detected by the algorithms >60 to >180 minutes in the accelerometry data (illustrated in Fig. 2, panel III, for the 60-min algorithm) on a minute-by-minute basis.

Analyses were performed using SAS^®^ Enterprise Guide^®^, version 4.3 (SAS Institute Inc., Cary, NC) as well as the R Statistical Programming Language for Windows, version 3.2.0^22^ and MS Access 2010/SQL via Microsoft Visual Basic for Application 7.0.

## Results

Characteristics of the study populations of ActivE (N = 47; men, 51.1%) and KORA (N = 559; men, 46.9%) are summarized in Table 1. According to the participants’ diary, the median number of NWT periods/24 h as well as median total length of NWT/24 h was similar between studies, (number, 0.8/24 h in ActivE, 0.9/24 h in KORA; length, 20.8 min/24 h in ActivE, 23.9 min/24 h in KORA) (Table 1). There were no significant differences in number of NWT periods/24 h or length of NWT/24 h between age groups of 20–39 years, 40–59 years, and 60+ years in ActivE (p = 0.36 and p = 0.88, respectively) and between age groups of <60 years and 60+ years in KORA (p = 0.54 and p = 0.96, respectively). Over the total time of assessment, 9.1% and 15.4% of the total NWT reported in the diary were >60 min in ActivE and KORA, respectively. Thus, in consequence, around 85% of the reported NWT periods were <60 minutes and thus not detectable by the algorithms. Common reasons for NWT reported were showering, personal hygiene, changing clothes etc. NWT periods >180 minutes made up <5% of all NWT reported. During waking, the proportion of reported NWT >60 to >180 minutes was comparable to the results seen over the total time of assessment, while during sleeping the proportions were considerably larger.

**Table 1:** Characteristics of the study populations of the ActivE and KORA FF4 study.

	ActivE study		KORA FF4 study	
**Characteristics of study population**				
number of participants, N	47		559	
men, %	51.1		46.9	
	**Median**	**IQR**	**Median**	**IQR**
age, years	43	(32–59)	58	(53–63)
height, cm	175.6	(165.8–180.1)	168.8	(162.0–176.8)
body weight, kg	78.3	(70.9–88.0)	78.9	(68.6–91.1)
BMI, kg/m²	25.4	(23.2–28.7)	27.2	(24.5–30.6)
**NWT based on diary** ^**a**^				
number of NWT periods/24 h	0.8	(0.6–1.0)	0.9	(0.6–1.0)
NWT/24 h, min	20.8	(12.4–30.3)	23.9	(13.6–44.5)
**Proportion of NWT periods**, **%**				
***total time of assessment*** ^**b**^				
>60 min		9.1		15.4
>90 min		4.5		8.7
>120 min		3.3		6.4
>150 min		2.0		5.1
>180 min		1.5		4.3
*waking* ^*c*^				
>60 min		8.6		13.1
>90 min		4.0		7.6
>120 min		2.6		5.2
>150 min		1.3		4.0
>180 min		0.5		3.2
*sleeping* ^*c*^				
>60 min		50.0		88.0
>90 min		50.0		84.0
>120 min		50.0		84.0
>150 min		50.0		84.0
>180 min		50.0		80.0

BMI, body-mass index; IQR, interquartile range; NWT, non-wear time. ^a^The total number of waking and sleeping phases were summed up per participant and divided by two to consider one waking and one sleeping phase as average 24 h.

^b^Analyses were conducted from the first to the last recorded time point of assessment in the diary.

^c^Waking and sleeping phases were derived from participants’ diary entries.

In ActivE, 657 waking and 656 sleeping phases of 47 participants were available for separate analyses. In KORA data of 559 participants were available for total time of assessment and 3,483 waking (546 participants) and 3,352 sleeping phases (551 participants) were included for separate analyses.

1. *Sensitivity and specificity of NWT algorithms* >*60 to* >*180 minutes based on accelerometry compared to diary*.

Overall, sensitivity and specificity to detect NWT in 24 h-accelerometry data were low for the 60-min algorithm in both studies (Table 2). Particularly, specificity was low for the total time of assessment (ActivE, 0.00; KORA, 0.08) and during sleeping (ActivE, 0.59; KORA, 0.77). In general, specificity increased with length of NWT algorithms. Sensitivity also increased with length of NWT algorithms for the total time of assessment. During sleeping phases, sensitivity already reached a maximum for the 60-min algorithm, being 1.00 in ActivE and 0.90 in KORA, respectively; sensitivity for longer NWT algorithms did not or only slightly decreased.

**Table 2:** Sensitivity and specificity: NWT algorithms >60 to >180 minutes using accelerometry versus diary.

NWT algorithm^a^	ActivE study (N = 47)				KORA FF4 study (N = 559)			
	number of NWT periods		sensitivity^b^	specificity^b^	number of NWT periods		sensitivity^b^	specificity^b^
	diary	accelerometry			diary	accelerometry		
*total time of assessment* ^*c*^								
>60 min	50	378	0.72	0.00	476	1,535	0.64	0.08
>90 min	25	81	0.80	0.16	269	420	0.70	0.57
>120 min	18	34	0.83	0.54	**199**	**284**	**0.76**	**0.74**
>150 min	11	17	0.82	0.78	156	223	0.72	0.79
>180 min	**8**	**10**	**0.88**	**0.92**	134	192	0.72	0.82
*waking* ^*d*^								
>60 min	47	75	0.70	0.93	369	531	0.65	0.91
>90 min	**22**	**34**	**0.76**	**0.97**	214	257	0.70	0.97
>120 min	14	17	0.71	0.99	**145**	**178**	**0.76**	**0.98**
>150 min	7	8	0.57	0.99	112	132	0.68	0.98
>180 min	3	3	0.67	1.00	91	108	0.68	0.99
*sleeping* ^*d*^								
>60 min	3	299	1.00	0.59	22	**868**	0.90	0.77
>90 min	**3**	**44**	**1.00**	**0.94**	**21**	**130**	**0.86**	**0.97**
>120 min	**3**	**16**	**1.00**	**0.98**	**21**	**81**	**0.86**	**0.98**
>150 min	**3**	**9**	**1.00**	**0.99**	**21**	**75**	**0.86**	**0.98**
>180 min	**3**	**6**	**1.00**	**1.00**	**20**	**66**	**0.85**	**0.99**

NWT, non-wear time.

^a^Algorithms were applied to diary and accelerometry data to check for self-reported NWT periods >60, >90, >120, >150, and >180 minutes or NWT periods based on consecutive acceleration zero-counts >60, >90, >120, >150, and >180 minutes, respectively.

^b^Periods with an overlap between NWT according to diary and accelerometry of ≥50% were included in this analysis; number of epochs with an overlap of <50% was: in ActivE, waking, 90-min algorithm: 1; in KORA FF4, total time, 60- to 180-min algorithm: 30, 7, 8, 5, and 4, respectively; in KORA FF4, waking, 60- to 180-min algorithm: 14, 4, 5, 2, and 1, respectively; in KORA FF4, sleeping, 60-min algorithm: 1.

^c^Analyses were conducted from the first to the last recorded time point of assessment in the diary.

^d^Waking and sleeping phases were derived from participants’ diary entries.

**bold**: algorithms showing high sensitivity and specificity in ActivE and KORA FF4, respectively.

For the total time of assessment, sensitivity and specificity were both high when applying the 180-min algorithm in ActivE and the 120-min algorithm in KORA, respectively.

During waking phases, both, sensitivity and specificity were high using the 90-min algorithm in ActivE and the 120-min algorithm in KORA, respectively.

During sleeping phases, both, sensitivity and specificity were high for NWT algorithms of >90 to >180 minutes of acceleration zero-counts with no or only slight differences in sensitivity and specificity between these algorithms in both studies.

2. *Overlap of accelerometry and diary NWT periods identified using the* >*60-min to* >*180-min algorithms*.

Results for the (minutes) of NWT detected by diary or accelerometry, and their overlap are shown in Table 3. Overall, in both studies the overlap was lowest when applying the 60-min algorithm: for the total time of assessment the length of NWT detected in both, *diary and accelerometry* contributed 18.0% in ActivE and 28.4% in KORA to all NWT minutes detected by either *diary*, *accelerometry*, or *both* (i.e., of potential total NWT). In contrast, the length of NWT detected in *diary only* (but not in accelerometry) contributed 4.2% in ActivE and 10.9% in KORA, whereas those detected by *accelerometry only* (but not in diaries) contributed 77.8% in ActivE and 60.7% in KORA, suggesting that a substantial proportion of NWT detected by accelerometry was false positively identified. This was mainly related to sleeping where about 90% of NWT time was detected by *accelerometry only* while it was about 40% during waking.

**Table 3:** Overlap of accelerometry and diary NWT periods identified using the >60-min to >180-min algorithms.

NWT algorithm^a^	ActivE study (N = 47)							
	diary NWT		accelerometry NWT		diary *and*/*or* accelerometry NWT minutes			
	total	periods	total	periods	potential total^b^	diary and accelerometry	diary only	accelerometry only
	min.	n	min.	n	min.	%	%	%
*total time of assessment* ^*c*^								
>60 min	7,891	50	34,127	378	35,612	18.0	4.2	77.8
>90 min	6,133	25	13,243	81	14,170	36.7	6.5	56.7
>120 min	5,400	18	8,301	34	9,156	49.6	9.3	41.0
>150 min	4,463	11	6,065	17	6,759	55.8	10.3	34.0
>180 min	3,983	8	4,903	10	5,436	63.5	9.8	26.7
*waking* ^*d*^								
>60 min	5,079	47	7,956	75	9,302	40.1	14.5	45.4
>90 min	3,321	22	4,914	34	5,702	44.4	13.8	41.8
>120 min	2,470	14	3,123	17	3,815	46.6	18.1	35.3
>150 min	1,533	7	1,934	8	2,465	40.6	21.5	37.8
>180 min	873	3	1,119	3	1,309	52.2	14.5	33.3
*sleeping* ^*d*^								
>60 min	1,257	3	24,382	299	24,418	5.0	0.1	94.9
>90 min	1,257	3	6,676	44	6,712	18.2	0.5	81.3
>120 min	1,257	3	3,832	16	3,868	31.6	0.9	67.5
>150 min	1,257	3	2,926	9	2,962	41.2	1.2	57.6
>180 min	1,257	3	2,401	6	2,437	50.1	1.5	48.4
	**KORA FF4 study (N = 559)**							
*total time of assessment* ^*c*^								
>60 min	83,642	476	189,666	1,535	212,912	28.4	10.9	60.7
>90 min	69,810	269	110,560	420	128,642	40.2	14.1	45.7
>120 min	62,806	199	95,794	284	111,616	42.1	14.2	43.7
>150 min	57,396	156	87,262	223	103,171	40.2	15.4	44.4
>180 min	53,933	134	81,623	192	96,786	40.1	15.7	44.3
*waking* ^*d*^								
>60 min	56,004	369	82,091	531	97,708	41.3	16.0	42.7
>90 min	45,447	214	61,874	257	74,082	44.9	16.5	38.7
>120 min	38,529	145	52,835	178	62,915	45.2	16.0	38.8
>150 min	34,359	112	46,430	132	56,822	42.2	18.3	39.5
>180 min	31,009	91	42,257	108	51,776	41.5	18.4	40.1
*sleeping* ^*d*^								
>60 min	9,331	22	86,690	868	88,080	9.0	1.6	89.4
>90 min	9,270	21	35,146	130	36,605	21.3	4.0	74.7
>120 min	9,270	21	30,204	81	31,663	24.7	4.6	70.7
>150 min	9,270	21	29,411	75	30,870	25.3	4.7	70.0
>180 min	9,108	20	27,923	66	29,381	26.0	5.0	69.0

NWT, non-wear time. ^a^Algorithms were applied to diary and accelerometry data to check for self-reported NWT periods >60, >90, >120, >150, and >180 minutes or NWT periods based on consecutive acceleration zero-counts >60, >90, >120, >150, and >180 minutes, respectively.

^b^Potential total NWT includes NWT which is NWT detected by either *diary*, *accelerometry*, or *both*.

^c^Analyses were conducted from the first to the last recorded time point of assessment in the diary.

^d^Waking and sleeping phases were derived from participants’ diary entries.

When focusing on the total time of assessment, the 180-min and 120-min algorithm that showed high sensitivity and specificity also showed the highest degree of overlap in NWT minutes detected by both, *diary and accelerometry* in ActivE (63.5%) and KORA (42.1%), respectively. Applying these algorithms, 26.7% and 43.7%, respectively, of total NWT time were detected by *accelerometry only*.

For waking phases, applying the most sensitive and specific 90-min and 120-min algorithm in ActivE and KORA, respectively, also resulted in a comparably high NWT minutes overlap, i.e. 44.4% and 45.2%, respectively. About 40% of total NWT time was detected by *accelerometry only* when using these algorithms.

During sleeping phases, using algorithms >90 to >180 minutes showing high sensitivity and specificity to detect NWT, the degree of overlap in NWT minutes identified by both, *diary and accelerometry* increased with length of the algorithm in both studies, from about 20% to 50% in ActivE and 26% in KORA. However, even with algorithms >180 minutes about 50% to 70% of the NWT were detected by *accelerometry only*.

3. *Overlap of any diary NWT with accelerometry NWT identified using the* >*60-min to* >*180-min algorithms*.

The overlap in NWT minutes identified by both, *diary and accelerometry* was low when using all reported NWT minutes from the diary (i.e., also less than 60 min) in comparison to accelerometry based NWT minutes using algorithms: for the total time of assessment the overlap ranged between 14.7% and 21.9% in ActivE and 21.8% and 26.7% in KORA (Table 4). The highest overlap was achieved in both studies when applying the 90-min and 120-min algorithm to identify accelerometry NWT. During sleeping, the overlap increased with algorithm length from 5.0% to a maximum of 49.4% in ActivE, and from 9.1% to 26.0% in KORA, respectively. Interestingly, when using the 60-min algorithm the overlap was highest during waking, i.e. 20.8% in ActivE and 27.3% in KORA, and decreased with increasing NWT algorithm length to 4.8% in ActivE and 17% in KORA, respectively.

**Table 4:** Overlap of any diary NWT with accelerometry NWT identified using the >60-min to >180-min algorithms.

NWT algorithm^a^	ActivE study (N = 47)							
	diary NWT		accelerometry NWT		diary *and*/*or* accelerometry NWT minutes			
	total	periods	total	periods	potential total^b^	diary and accelerometry	diary only	accelerometry only
	min.	n	min.	n	min.	%	%	%
*total time of assessment* ^*c*^								
>60 min	17,231	551	34,127	378	44,782	14.7	23.8	61.5
>90 min	17,231	551	13,243	81	25,136	21.2	47.3	31.4
>120 min	17,231	551	8,301	34	20,949	21.9	60.4	17.7
>150 min	17,231	551	6,065	17	19,404	20.1	68.7	11.2
>180 min	17,231	551	4,903	10	18,662	18.6	73.7	7.7
*waking* ^*d*^								
>60 min	14,390	546	7,956	75	18,496	20.8	57.0	22.2
>90 min	14,390	546	4,914	34	16,671	15.8	70.5	13.7
>120 min	14,390	546	3,123	17	15,697	11.6	80.1	8.3
>150 min	14,390	546	1,934	8	15,199	7.4	87.3	5.3
>180 min	14,390	546	1,119	3	14,804	4.8	92.4	2.8
*sleeping* ^*d*^								
>60 min	1,302	6	24,382	299	24,462	5.0	0.3	94.7
>90 min	1,302	6	6,676	44	6,756	18.1	1.2	80.7
>120 min	1,302	6	3,832	16	3,912	31.3	2.0	66.7
>150 min	1,302	6	2,926	9	3,006	40.8	2.7	56.7
>180 min	1,302	6	2,401	6	2,481	49.4	3.2	47.5
	**KORA FF4 study (N = 559)**							
*total time of assessment* ^*c*^								
>60 min	142,640	3,089	189,666	1,535	270,588	22.8	29.9	47.3
>90 min	142,640	3,089	110,560	420	199,817	26.7	44.7	28.6
>120 min	142,640	3,089	95,794	284	190,185	25.4	49.6	25.0
>150 min	142,640	3,089	87,262	223	186,905	23.0	53.3	23.7
>180 min	142,640	3,089	81,623	192	184,194	21.8	55.7	22.6
*waking* ^*d*^								
>60 min	112,233	2,811	82,091	531	152,628	27.3	46.2	26.5
>90 min	112,233	2,811	61,874	257	139,417	24.9	55.6	19.5
>120 min	112,233	2,811	52,835	178	135,672	21.7	61.1	17.3
>150 min	112,233	2,811	46,430	132	133,561	18.8	65.2	16.0
>180 min	112,233	2,811	42,257	108	131,995	17.0	68.0	15.0
*sleeping* ^*d*^								
>60 min	9,488	25	86,690	868	88,166	9.1	1.7	89.2
>90 min	9,488	25	35,146	130	36,752	21.4	4.4	74.2
>120 min	9,488	25	30,204	81	31,810	24.8	5.0	70.2
>150 min	9,488	25	29,411	75	31,017	25.4	5.2	69.4
>180 min	9,488	25	27,923	66	29,691	26.0	6.0	68.0

NWT, non-wear time. ^a^Algorithms were applied only to accelerometry data to check for periods based on consecutive acceleration zero-counts >60, >90, >120, >150, and >180 minutes, respectively.

^b^Potential total NWT includes NWT which is NWT detected by either *diary*, *accelerometry*, or *both*.

^c^Analyses were conducted from the first to the last recorded time point of assessment in the diary.

^d^Waking and sleeping phases were derived from participants’ diary entries.

Over the total time of assessment the proportion of NWT minutes detected in *diary only* increased with increasing algorithm length in both studies, i.e. from 23.8% to 73.7% in ActivE and from 29.9% to 55.7% in KORA. Comparing waking and sleeping revealed that this is mainly due to NWT during waking where up to 92.4% (ActivE) and 68.0% (KORA) were detected in *diary only*.

The proportion of NWT minutes detected by *accelerometry only* decreased in both studies with increasing algorithm length, for total time of assessment, waking, and sleeping. Particularly during sleeping the proportion was very high, about 90% for the 60-minute algorithm and still about 70% for the 120-minute algorithm.

Considering the algorithms that showed highest sensitivity and specificity (Table 2) during waking phases, the 90-min and 120-min algorithm, the overlap in NWT minutes detected in *diary and accelerometry* was about 20% only. Assessing the same for the sleeping phases the overlap in NWT minutes between *diary and accelerometry* was between 20% and 50% when applying NWT algorithms >90 to >180 minutes to accelerometry data.

## Discussion

In this study, we tested the suitability of different algorithms to detect NWT in 24 h-accelerometry in two independent epidemiological studies in the general adult population. Overall, periods longer than 60 minutes made up only 10–15% of all NWT periods reported, whereas around 85–90% of total NWT minutes were spent in periods <60 minutes. This indicates that a considerable amount of NWT minutes was missed by all algorithms tested. When using the 60-min algorithm sensitivity and specificity to detect NWT based on 24 h-accelerometry was limited in comparison to diary information. The same was true when analyzing the overlap in NWT minutes between diary and accelerometry. Further, applying the 60-min algorithm resulted in high rates of false positive NWT detection based on accelerometry data. Algorithms between >90 to >180 minutes showed higher sensitivity, specificity, and larger overlap between NWT based on diary and accelerometry. However, we still found considerable differences when comparing diary and accelerometry derived NWT using algorithms regarding its overlap. Thus, our data suggests that the 60-min algorithm is less suitable for NWT detection in 24 h-accelerometry. Among the different algorithms assessed here, the 120-min algorithms seems to be a compromise between high sensitivity, specificity, large overlap, and the detection of as much NWT as possible. This holds true for continuous 24 h-accelerometry data as well as for waking and sleeping phases only.

Correct and precise detection of NWT periods is important for unbiased accelerometry based estimation of habitual physical activity. When NWT periods are unknowingly included in activity estimates, they lead to an overestimation of the time spent in the lowest physical activity intensity^23^. Conversely, when true wear periods without movements are classified as NWT, the subsequent exclusion of this time leads to an underestimation of sedentary behavior and overestimation of relative time in physical activity; this effect was shown in a study on sedentary behavior in children^24^.

The most common approach to identify NWT is to define >60 consecutive minutes of zero-counts as an objective criterion and was originally developed for accelerometry conducted during waking hours only^9, 15^. However, 24 h-accelerometry is increasingly applied, particularly in large-scale epidemiological studies, since it provides a comprehensive picture of activity habits encompassing also periods in between being awake and asleep and further reduces the likelihood of forgetting to put on the accelerometer after sleeping. Thus, we assessed algorithms to detect NWT periods of >60 to >180 minutes over the total time of assessment as well as separately for waking and sleeping phases. In general, as one may have expected, we detected fewer NWT periods in diary and accelerometry data the longer the NWT definition was.

*Waking phases*. Although the common 60-min algorithm was developed for waking hours, our data showed that only a small part of the total NWT periods during waking phases reported in diaries is actually longer than 60 minutes, indicating that a substantial proportion of NWT will be missed with this algorithm. Further, sensitivity and especially specificity of the 60-min algorithm was low. In addition, the overlap in NWT minutes identified by both, *diary and accelerometry* was lowest for the 60-min algorithm and the rate of false positively detected NWT highest comparing accelerometry and diary (Table 3). We found higher sensitivity, specificity, and larger overlap for the 90- and 120-min algorithm. Several previous studies indicate that NWT algorithms longer than 60 minutes improve NWT detection during waking hours. Peeters *et al*. recommended a 90-min algorithm that has already been applied in few field studies^7, 25, 26^. Choi *et al*. showed lower rates of NWT misclassification for a 90-min than for a 60-min algorithm^27, 28^. Hutto *et al*. found 60- and 90-min algorithms to substantially overestimate NWT and underestimate sedentary time and suggested a 120-min NWT algorithm^29^. Finally, Oliver *et al*. concluded that a 180-min algorithm is most suitable for NWT detection during waking hours; however this study was focused on a sedentary population^30^. Nevertheless, even when we applied the ‘best’ algorithms according to our analyses, only about 20% of all NWT reported regardless of their length were detected using accelerometry (Table 4).

*Sleeping phases*. In contrast to waking phases, periods with no detectable movement over 60 minutes or longer are likely to occur during sleeping phases. However, studies investigating NWT during sleeping phases are scarce. In our study, a minority of all NWT reported in the diaries occurred during sleeping phases and those occurred were mainly longer than all algorithms applied. Thus, sensitivity was generally high. However, specificity of the 60-min algorithm was substantially lower compared to the longer NWT algorithms, indicating that wearing time during sleep is often falsely assigned as NWT; indeed the proportion of NWT minutes false positively detected by *accelerometry only* was remarkably higher for the 60-min than for all longer NWT algorithms. At the same time, overlap of NWT minutes was extremely low for the 60-min algorithm but achieved even with the 180-min algorithms only 50% and 26% in ActivE and KORA, respectively. Specificity during sleeping phases was slightly higher in KORA than in ActivE. This might be explained by the fact that KORA participants wore the accelerometer on the wrist during sleeping phases; moving the arm/hand during sleeping is more likely and thus the probability of false negative NWT detection lower for wrist- than for waist-worn devices^27^. Taken our results into account and considering the fact that very few NWT periods occur during sleeping the algorithms tested have to be valued with caution as they might introduce a bias that is larger than just assuming no NWT during sleeping.

*Total time of assessment.* In our analysis, the 60-min algorithm showed moderate sensitivity and extremely low specificity to detect NWT periods when analyzing 24 h-accelerometry data. The high degree of false positively detected NWT during sleeping might substantially contribute to the extremely low specificity of NWT detection in 24 h-accelerometry data; however, surprisingly, even during waking phases, the 60-min algorithms failed to reliably detect NWT. Further, we observed the overlap of the length of NWT detected by both, *diary and accelerometry* to be lowest and the proportion of NWT detected by *accelerometry only* to be highest applying the 60-min NWT algorithm over the total time of assessment, indicating only little agreement between accelerometry and diary based NWT. Again, we found considerable limitations of this algorithm during waking and especially sleeping resulting in poor overlap found for the total time of assessment. Consequently, our data indicate that the 60-min algorithm is less suitable for NWT detection in continuous 24 h-accelerometry data. Falsely classifying longer periods of true wear time without activity as NWT might lead to a substantial underestimation of sedentary time (including sleeping) and overestimation of relative time in activity. Among those tested, the algorithms that had high sensitivity, specificity, and degree of overlap in NWT minutes over the total time of assessment were the 180- and 120-minutes algorithm in ActivE and KORA, respectively; however, still a large proportion of reported NWT was not detected when applying these algorithms. Accordingly, the detection of shorter periods of NWT is limited.

In our studies, short NWT periods (i.e., <60 minutes) accounted for the majority of all NWT periods, which is comparable to the study by Oliver *et al*. assessed during waking hours^30^. Further, our data show that even with the ‘best’ of the tested algorithms, most of these NWT minutes were not detected in 24 h-accelerometry as compared to diary data. In case the accelerometer was removed and physical activity conducted during these periods of NWT, their inclusion would result in an underestimation of physical activity. However, the main reasons for NWT reported in the diaries of the ActivE study were showering and changing clothes, and, as such, not related to substantial physical activity. We therefore speculate that inclusion of these shorter periods of NWT is likely to have only little effect on physical activity estimates.

When assessing sensitivity and specificity of the NWT algorithms, we considered the diaries to be the ‘gold standard’ reference. This seems reasonable since we expected participants to report the occurrence of NWT quite accurately. However, we speculate that the reported information on the length of NWT might be less precise than information derived from accelerometers, e.g., participants may tend to round or estimate time points. Thus, there may be the dilemma that while the diary may be the gold standard to detect the presence or absence of true NWT, accelerometers may be more valid to estimate the exact time of its beginning, ending, and length.

A strength of our study was that we analyzed two independent studies on the general adult population from different regions in Germany covering a broad spectrum of participants’ characteristics including highly motivated, voluntary, as well as population-based participants. In contrast to most other studies, we had 24 h-accelerometry as well as diary data recorded over 1–2 weeks, allowing a differentiated analysis for total time of assessment and for waking and sleeping phases. Nevertheless, our study has some limitations. First of all it’s limited to adults. Although we had two independent studies that should cover the range of characteristics seen in the general population, specific phenotypes, e.g. extreme obesity, advanced cardiovascular or respiratory diseases, and narrower, younger, or older ages might show other frequencies and durations of (non−) moving phases as well as different compliance to wearing the accelerometer. Further, participants of the ActivE study constantly wore the accelerometer on the right hip, while KORA participants changed the devices from the hip to the wrist for sleeping; however, absolute values and relative differences between algorithms were similar between both studies. Thus, this methodological aspect in the comparative analysis might not be substantial^31^. Additionally, wear time periods were defined as absence of any NWT; thus the specificity for the total time of assessment cannot directly be compared to the specificity of waking or sleeping phases, and the absolute values should be interpreted cautiously. Further, we assumed the participants’ diary data as ‘gold standard’ and any misreporting of our study participants may therefore dilute our findings. Finally, a general limitation of any NWT algorithm >60 minutes is that shorter NWT are not detectable at all but made up a substantial part of NWT. Thus, further studies on alternative approaches to detect NWT <60 minutes are warranted to enable a reliable and comprehensive NWT detection.

In conclusion, our data indicate that the 60-min algorithm is not suitable to detect NWT in 24 h-accelerometry data in epidemiological studies. This was due to a high rate of false positively detected NWT by accelerometry. NWT detection should be valued with caution during sleeping given the weak performance of all assessed algorithms and the low number of NWT during sleeping. All algorithms assessed may miss a substantial proportion of short NWT, which made up the vast majority of all NWT reported. Even the most sensitive and specific algorithms still false positively identified a large proportion of NWT based on accelerometry. Although each has limitations, among the different algorithms assessed here, applying the 120-min algorithm seems to be a compromise between accuracy and detection of as much NWT as possible in 24 h-accelerometry data.

## References

[1] US Department Of Health and Human Services Physical Activity and Health: A Report of the Surgeon General. U.S. GPO, Washington: Atlanta, GA: U.S.Department of Health and Human Services, Centers for Disease Control and Prevention, National Center for Chronic Disease Prevention and Health Promotion 81–172p (1996).

[2] World Health Organization. Global Recommendations on Physical Activity for Health. WHO Press, World Health Organization, Geneva, Switzerland http://whqlibdoc.who.int/publications/2010/9789241599979_eng.pdf?ua=1 (accessed 4 July 2016) (2010).

[3] World Health Organization. Global Health Risks - Mortality and burden of disease attributable to selected major risks. http://www.who.int/iris/handle/10665/44203 (accessed 22 July 2016) (2009).

[4] Kwak L, Proper KI, Hagstromer M, Sjostrom M (2011). The repeatability and validity of questionnaires assessing occupational physical activity–a systematic review. Scand J Work Environ Health..

[5] Prince SA (2008). A comparison of direct versus self-report measures for assessing physical activity in adults: a systematic review. Int J Behav Nutr Phys Act..

[6] Sallis JF (2016). Physical activity in relation to urban environments in 14 cities worldwide: a cross-sectional study. Lancet..

[7] Keevil VL (2016). Objective Sedentary Time, Moderate-to-Vigorous Physical Activity, and Physical Capability in a British Cohort. Med Sci Sports Exerc..

[8] Keadle SK, Shiroma EJ, Freedson PS, Lee IM (2014). Impact of accelerometer data processing decisions on the sample size, wear time and physical activity level of a large cohort study. BMC Public Health..

[9] Troiano RP (2008). Physical Activity in the United States Measured by Accelerometer. Med Sci Sports Exerc..

[10] Warren JM (2010). Assessment of physical activity - a review of methodologies with reference to epidemiological research: a report of the exercise physiology section of the European Association of Cardiovascular Prevention and Rehabilitation. Eur J Cardiovasc Prev Rehabil.

[11] German National Cohort (GNC) Consortium (2014). The German National Cohort: aims, study design and organization. Eur J Epidemiol..

[12] Pedisic Z, Bauman A (2015). Accelerometer-based measures in physical activity surveillance: current practices and issues. Br J Sports Med..

[13] Ekblom-Bak E (2016). SCAPIS Pilot Study: Sitness, Fitness and Fatness - Is Sedentary Time Substitution by Physical Activity Equally Important for Everyone’s Markers of Glucose Regulation?. J Phys Act Health..

[14] Matthews CE (2008). Amount of time spent in sedentary behaviors in the United States, 2003–2004. Am J Epidemiol..

[15] National Cancer Institute. SAS programs for analyzing NHANES 2003–2004 accelerometer data. http://riskfactor.cancer.gov/tools/nhanes_pam/ (accessed December 11 2016).

[16] Holle R, Happich M, Löwel H, Wichmann HE, Group MKS (2005). KORA–a research platform for population based health research. Gesundheitswesen..

[17] Aadland E, Ylvisåker E (2015). Reliability of the Actigraph GT3X+ Accelerometer in Adults under Free-Living Conditions. PLoS One.

[18] Santos-Lozano A (2012). Technical variability of the GT3X accelerometer. Med Eng Phys..

[19] Sasaki JE, John D, Freedson PS (2011). Validation and comparison of ActiGraph activity monitors. J Sci Med Sport..

[20] Luzak A (2017). Physical activity levels, duration pattern and adherence to WHO recommendations in German adults. PLoS One..

[21] Mayer, D. *Essential Evidence-Based Medicine - Second Edition* New York, USA (Cambridge University Press, 2010).

[22] R Core Team. *R*: *A language and environment for statistical computing*. *R Foundation for Statistical Computing* Vienna, Austria http://www.R-project.org/(accessed April 4, 2016) (2015).

[23] Janssen X (2015). Objective measurement of sedentary behavior: impact of non-wear time rules on changes in sedentary time. BMC Public Health..

[24] Meredith-Jones K, Williams S, Galland B, Kennedy G, Taylor R (2016). 24 h Accelerometry: impact of sleep-screening methods on estimates of sedentary behaviour and physical activity while awake. J Sports Sci..

[25] Peeters G, van Gellecum Y, Ryde G, Farias NA, Brown WJ (2013). Is the pain of activity log-books worth the gain in precision when distinguishing wear and non-wear time for tri-axial accelerometers?. J Sci Med Sport..

[26] Aguilar-Farias, N., Brown, W. J. & Peeters, G. M. ActiGraph GT3X+ cut-points for identifying sedentary behaviour in older adults in free-living environments. *J Sci Med Sport.* **17**, 293–299, doi:10.1016/j.jsams.2013.07.002 (2014). 10.1016/j.jsams.2013.07.002 23932934

[27] Choi L, Ward SC, Schnelle JF, Buchowski MS (2012). Assessment of wear/nonwear time classification algorithms for triaxial accelerometer. Med Sci Sports Exerc..

[28] Choi L, Liu Z, Matthews CE, Buchowski MS (2011). Validation of accelerometer wear and nonwear time classification algorithm. Med Sci Sports Exerc.

[29] Hutto B (2013). Identifying accelerometer nonwear and wear time in older adults. Int J Behav Nutr Phys Act.

[30] Oliver M, Badland HM, Schofield GM, Shepherd J (2011). Identification of accelerometer nonwear time and sedentary behavior. Res Q Exerc Sport..

[31] Dieu, O. *et al*. Physical activity using wrist-worn accelerometers: comparison of dominant and non-dominant wrist. *Clin Physiol Funct Imaging*, doi: 10.1111/cpf.12337 (2016). 10.1111/cpf.12337 26749436

